# Kruppel-Like Factor 4 (KLF4) and its Regulation on Mitochondrial Homeostasis

**DOI:** 10.4172/2157-7633.1000436

**Published:** 2018-09-14

**Authors:** Brian Tung, Shuli Xia

**Affiliations:** 1 Hugo W. Moser Research Institute at Kennedy Krieger, Baltimore, Maryland, USA; 2 Department of Neurology, Johns Hopkins University School of Medicine, Baltimore, Maryland, USA

**Keywords:** Kruppel-like factor 4, Mitochondria, Mitochondrial homeostasis, Stress, Heart failure, Glioblastoma

## Abstract

Mitochondria are vital organelles that supply ATP and other energy metabolites to meet the bioenergetics demands of the cell. In environments of stress or increased energy requirement, mitochondria are highly dynamic and can undergo biogenesis, fusion/fission, or autophagy. The transcription factor family, Kruppel-Like Factor (KLF), is necessary to carry out normal cellular processes from proliferation to differentiation. Recently, its importance in metabolic homeostasis in various tissue types has gained much attention. A handful of evidence supports KLF4’s involvement in regulating mitochondrial homeostasis in both healthy and cancer cells. In this review, we aim to summarize the available literature that demonstrates KLF4’s ability to modulate the mitochondrial life cycle in:

- Cardiac tissue, in which KLF4-knockdown subsequently leads to Heart Failure (HF), and
- Glioblastoma (GBM), where its expression promotes extensive mitochondrial fusion and offers mild cell protection under serum-deprivation

## Introduction

KLFs are a family of zinc finger-containing transcription factors that are critically involved in cellular processes such as proliferation, migration, differentiation, and pluripotency [1]. The 18 KLFs to date encoded in the human genome regulate a diverse array of biological functions under both physiology and pathophysiology conditions in many organs including the lung, liver, heart, brain, and skeletal muscle [2]. Recently, KLF4 has gained much attention not only due to its multifaceted roles in normal and cancer cells, but also due to its key function as one of the four transcription factors required for induced pluripotency. Specifically, KLF4 has been demonstrated to mediate gene regulation in a myriad of organ tissues. KLF4 serves as transcriptional activators or repressors that preferentially bind to GC/GT-rich regions with a core consensus sequence of C(A/G)CCC [3].

Despite emerging studies that highlight the paramount role of KLFs in cellular processes, there is limited research that relates the significance of KLFs and cellular metabolism. Mitochondria, central players in energy metabolism, have long been recognized as semi-autonomous organelles that supply ATP and other energy metabolites to meet the bioenergetics demands of the cell. Mitochondrial morphology and number are regulated through 3 key mechanisms: biogenesis, dynamics (fusion/fission), and selective autophagy (mitophagy). Mitochondrial biogenesis is required for cell growth to satisfy increased energy demands and is induced in response to limited nutrient availability and oxidative stress [4]. To maintain metabolic homeostasis, mitochondria undergo dynamic mechanical changes comprised of both fusion and fission of the mitochondrial architecture, whereas dysregulation of mitochondrial dynamics have been associated with metabolic impairment and disease [5,6]. As evolutionarily conserved behaviors that allow cells to respond to environmental stressors such as nutrient deficiency and intracellular stress, mitochondrial fusion and fission act as opposing phenomena with fusion favoring an interconnected network of mitochondria in highly metabolic cells, whereas fission is characterized by fragmented mitochondria for transport to loci of high energy expenditure or for mitotic distribution [5,7]. Consequently, mitochondrial fusion and fission are essential for the maintenance of mitochondrial homeostasis by replacing damaged contents or repairing through a process of continuous DNA exchange called complementation [8]. Moreover, optimal mitochondrial quality is maintained through mitophagy, a form of selective autophagy that sequesters defective mitochondria for lysosomal degradation [9].

Burgeoning evidence has implicated the essential role of KLF family members on mitochondrial maintenance from biogenesis, dynamics, and mitophagy in several organ systems [10,11]. In this review, we will highlight the direct role of KLF4 on mitochondrial activity on specific aspects of the cardiovascular system and nervous system.

## KLF4 as a critical regulator in cardiac mitochondrial homeostasis

Despite comprising only roughly 0.5% of one’s body weight, the heart consumes approximately 8% of all available ATP [12]. The heart, one of the most metabolically active organs in the body, requires a vast number of mitochondria to supply energy metabolites in order to maintain function. In fact, mitochondria comprise up to roughly a third of the heart’s cell volume in mammals [13]. Cardiac mitochondria are prolific organelles that supply ATP for the beating heart and its dysfunction is widely noted in the pathophysiology of HF.

HF is a multifaceted complex syndrome that is characterized by the heart’s inability to supply adequate blood to meet the circulatory and metabolic requirements of the body. This mechanical dysfunction of the heart has been linked to pathogenic factors including defects in the cardiac cycle, impaired signal transduction, and neurohormonal dysregulation [14]. Several studies have noted bioenergetic impairment in HF by way of decreased ATP availability, phosphocreatine (PCr), and PCr/ATP ratio [12].

Liao, et al. [15] published an article that highlights KLF4’s vital role in maintaining mitochondrial biogenesis and homeostasis in the heart. Previously, the group genetically modified mice resulting in cardiac-specific KLF4 deficiency, then subsequently subjected them to Transaortic Constriction (TAC), a procedure that challenges the heart with a greater pressure gradient against which it must pump, thereby artificially creating increased cardiac metabolic demand [16]. This stress challenge conferred a high susceptibility to HF and death. Compared with control, significant reductions in ATP levels within the myocardium and increased Reactive Oxygen Species (ROS) production subsequent to TAC were noted in cardiac-specific KLF4 deficient mice. Histology revealed extensive mitochondrial dysfunction and damage such as disarray, degeneration, and fragmentation after TAC in KLF4-deficient mice. Freshly isolated hearts from KLF4 deficient mice exhibited reduced respiratory rates and defective mitochondria at either the Kreb’s cycle or electron transport chain level. Apart from impaired mitochondrial function, the morphology of mitochondria trended towards a more fragmented state that suggested injury and degeneration over a period of time. Moreover, KLF4-deficiency in prenatal hearts prevented normal mitochondrial biogenesis, ultimately leading to HF and subsequent death.

Liao, et al. [15] were able to mechanistically relate KLF4 and its regulation of the mitochondria, building upon well-established literature of an interconnected network of transcription factors such as nuclear receptors (i.e., Estrogen-Related Receptors (ERRs), PPARγ Coactivator 1 family (PGC-1)) and nuclear respiratory receptors that act on nuclear and mitochondrial genome [17]. In fact, KLF4 interacts with ERRα and PGC-1 to form a tripartite protein complex that binds to promoter regions of mitochondrial DNA. Notably, knockdown of KLF4 in cardiac cells inhibited promoter transactivation of mitochondrial DNA.

The accumulation of fragmented mitochondria in KLF4-deficient cardiac cells warranted further investigation. An outstanding question that remained was whether KLF4 loss-of-function handicaps the process of autophagy. Indeed, KLF4 regulates genes involved in autophagy, thus providing equipoise to its equally significant role in mitochondrial biogenesis. Overall, the work from Liao, et al. has revealed a KLF4-dependent metabolic signature in mitochondrial gene expression involved in biogenesis and dynamics [15].

## KLF4 regulates mitochondrial dynamics in GBM

GBM, a highly malignant grade IV primary brain tumor, confers a dismal median survival of less than 2 years despite modern treatment modalities consisting of tumor resection and concomitant chemotherapy and radiation [18]. Recurrence of tumor due to tumor cell infiltration and Cancer Stem Cells (CSCs) is a herculean challenge that current therapies fail to adequately address. With uncontrolled proliferation as one of the hallmarks of this disease, cancer cells possess dysfunctional cellular metabolism that deliver energy metabolites for tumor maintenance and growth [19].

Essential for tumor cellular metabolism, mitochondrial function has been shown to be altered in a myriad of brain tumors like GBM [20,21]. Morphological changes in mitochondria due to cristolysis, swelling, and abnormal cycles of fusion and fission impart a heterogeneous mix of mitochondria well-documented in gliomas [22]. Recorded in several tumor types, CSCs are increasingly dependent on mitochondria [23]. In fact, defunct mitochondria in cancer may promote enhanced proliferation, escape from apoptosis, and switch from a state of catabolism to anabolism (Warburg effect), further supporting tumor initiation and infiltration [24]. Thus, mitochondrial targeting using agents like tetracyclines, which has known side effects of mitochondrial toxicity, with concomitant standard therapies, may represent a novel approach to better treat GBM [25].

A few research groups, including ours, have described the involvement of KLFs in GBM. Serving as both activators and repressors, KLF6 and KLF9 are two such members of the KLF family that attenuate GBM’s malignant phenotypes by inhibiting growth potential [26–28]. Wan and colleagues [29] recently discovered that KLF4 interacts with methylated DNA to activate RhoC expression via chromatin remodeling, in turn promoting GBM adhesion and migration, two hallmark tumor characteristics that make combatting GBM difficult.

Recently, our group contributed to the body of literature implicating the important role of KLF4 on the mitochondrial homeostasis. In the work from Wan, et al., genome-wide RNA sequencing revealed KLF4’s role in both the up- and down-regulation of mitochondrial genes, suggesting its direct involvement in mitochondrial homeostasis [29]. In the recent work from Wang, et al. [30], we observed extensive flux in mitochondrial architecture, consistent with increased mitochondrial fusion in KLF4-expressing GBM tumor cells. Mitochondrial dynamic changes did not substantially influence mitochondrial function under basal conditions when cells were cultured in full serum. However, KLF4 significantly increased the reserve respiratory capacity of GBM cells when challenged with pharmacological agents that affect mitochondrial function. Reserve (or spare) respiratory capacity is a measure of a cell’s capability to meet metabolic demands and its ability to respire to the theoretical maximum under stress conditions. Further, KLF4 expression promoted a significant increase in oxygen consumption rate under mitochondrial stress conditions. This unusually discovery of KLF4 function suggests its biological implication under stress conditions such as nutrition-deprivation. Indeed, KLF4 expression in GBM cells attenuated cell death following serum withdrawal by providing a metabolic advantage. KLF4-expressing cells portrayed evidence of extensive mitochondrial fusion that may explain the delayed cell death.

Mechanistically, KLF4 did not directly regulate the three major modulators of mitochondrial fusion: Mfn1, Mfn1, and Opa-1. However, for the first time, KLF4 has been found to interact with members of the Guanine Exchange Factor (GEF) family to modulate mitochondrial dynamics [30], including Neuronal Guanine Nucleotide Exchange Factor (NGEF) and Rho/Rac Guanine Nucleotide Exchange Factor 2 (ARHGEF2).

## Conclusion

**Figure 1:**
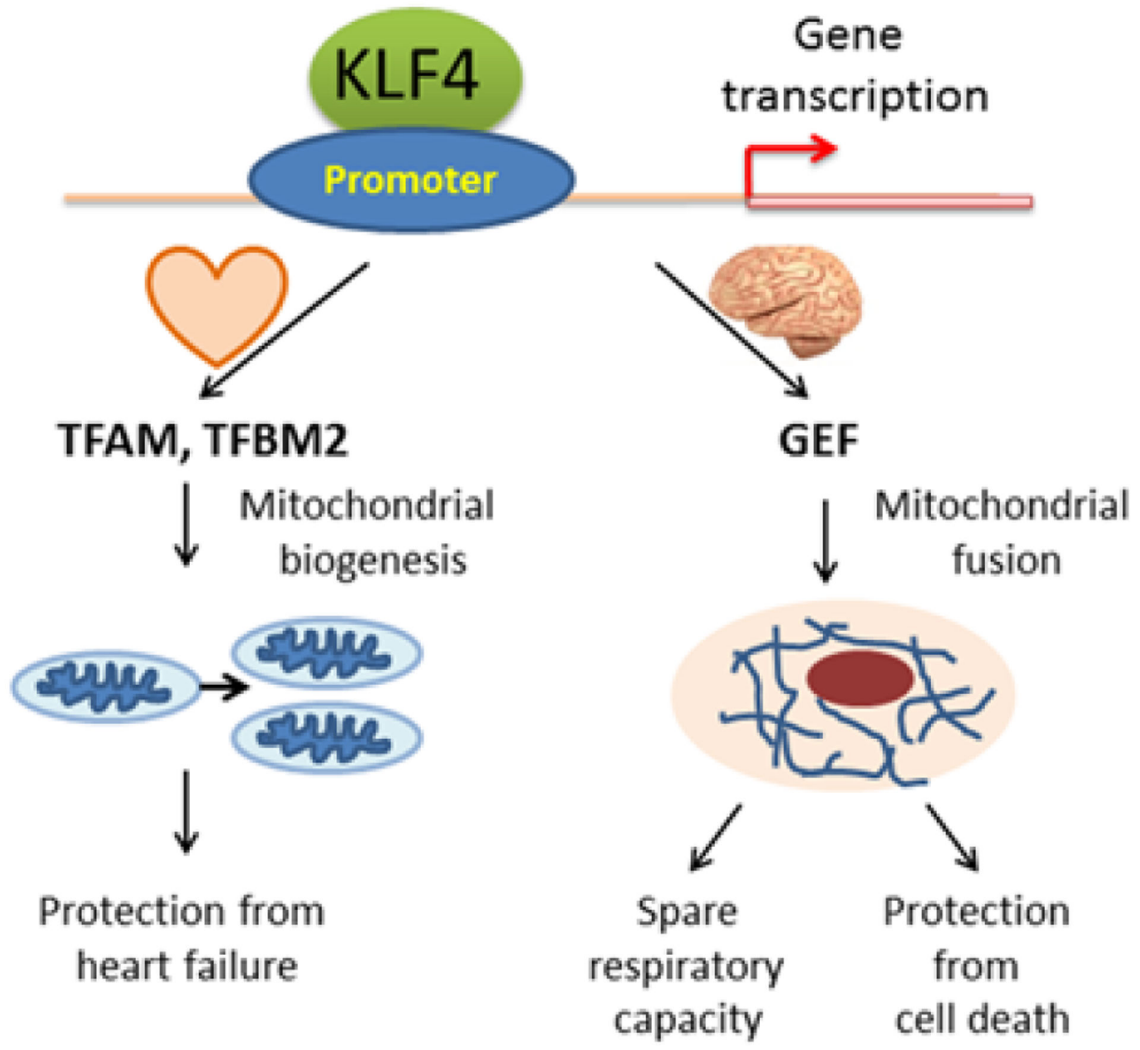
KLF4 regulates mitochondrial homeostasis.

Much of mitochondrial regulation is still unknown. Yet, emerging evidence identifies KLF4 as a major key player in mitochondrial homeostasis in both healthy and cancerous tissues. In the normal heart, KLF4-deficiency leads to downstream mitochondrial fragmentation, abnormal cycles of biogenesis, and autophagy, and eventual hypertrophic cardiac muscle, resulting in HF and death. In GBM, not only is KLF4 essential for mitochondrial fusion and cell protection in mitochondrial-stress environments, but also targets GEFs to serve as effectors for these morphological and functional changes (Figure 1). Despite the pooling studies that corroborate KLF4’s unquestionable impact on mitochondrial function in normal and cancer cells, more research is needed to better gain mechanistic insights. With new excitement to further investigate the role of KLFs in mitochondrial function, it is with optimism that current and future research can pave the way for medical advances in heart regeneration to eradication of brain tumors.

## Abbreviations:

KLF: Kruppel-Like Factor
HF: Heart Failure
GBM: Glioblastoma
PGC: PPARγ Coactivator
CSCs: Cancer Stem Cells

## References

[1] McConnell BB, Yang VW (2010) Mammalian Kruppel-like factors in health and diseases. Physiol Rev 90: 1337–1381. 20959618 10.1152/physrev.00058.2009 PMC2975554

[2] Pollak NM, Hoffman M, Goldberg IJ, Drosatos K (2018) Kruppel-Like Factors. JACC Basic to Transl Sci 3: 132–156. 10.1016/j.jacbts.2017.09.001 PMC5985828 29876529

[3] Kaczynski J, Cook T, Urrutia R (2003) Sp1- and Kruppel-like transcription factors. Genome Biol 4: 1–8. 10.1186/gb-2003-4-2-206 PMC151296 12620113

[4] Boland ML, Chourasia AH, Macleod KF (2013) Mitochondrial dysfunction in cancer. Front Oncol 3: 292. 24350057 10.3389/fonc.2013.00292 PMC3844930

[5] Wai T, Langer T (2016) Mitochondrial dynamics and metabolic regulation. Trends Endocrinol Metab 27: 105–117. 26754340 10.1016/j.tem.2015.12.001

[6] Archer SL (2013) Mitochondrial dynamics – mitochondrial fission and fusion in human diseases. N Engl J Med 369: 2236–2251. 24304053 10.1056/NEJMra1215233

[7] Westermann B (2010) Mitochondrial fusion and fission in cell life and death. Nat Rev Mol Cell Biol 11: 872–884. 21102612 10.1038/nrm3013

[8] Sato A, Nakada K, Hayashi JI (2009) Mitochondrial complementation preventing respiratory dysfunction caused by mutant mtDNA. Biofactors 35: 130–137. 19449440 10.1002/biof.14

[9] Kubli DA, Gustafsson AB (2012) Mitochondria and mitophagy: The yin and yang of cell death control. Circ Res 111: 1208–1221. 23065344 10.1161/CIRCRESAHA.112.265819 PMC3538875

[10] Zheng X, Bi C, Brooks M (2015) Kruppel-Like Factors: Crippling and Uncrippling Metabolic Pathways. Anal Chem 25: 368–379.

[11] Jang C, Arany Z (2015) Mitochondria Cripple without Kruppel. Trends Endocrinol Metab 26: 587–589. 26404663 10.1016/j.tem.2015.08.004 PMC4797320

[12] Brown DA, Perry JB, Allen ME, Sabbah HN, Stauffer BL (2016) Expert consensus document: Mitochondrial function as a therapeutic target in heart failure. Nat Rev Cardiol 14: 238–250. 28004807 10.1038/nrcardio.2016.203 PMC5350035

[13] Schaper J, Meiser E, Stammler G (1985) Ultrastructural morphometric analysis of myocardium from dogs, rats, hamsters, mice, and from human hearts. Circ Res 56: 377–391. 3882260 10.1161/01.res.56.3.377

[14] Rosca MG, Hoppel CL (2010) Mitochondria in heart failure. Cardiovasc Res 88: 40–50. 20668004 10.1093/cvr/cvq240 PMC3025720

[15] Liao X, Zhang R, Lu Y, Prosdocimo DA, Sangwung P, (2015) Kruppel-like factor 4 is critical for transcriptional control of cardiac mitochondrial homeostasis. J Clin Invest 125: 3461–3476. 26241060 10.1172/JCI79964 PMC4588311

[16] Liao X, Haldar SM, Lu Y, Jeyaraj D, Paruchuri K, (2010) Kruppel-like factor 4 regulates pressure-induced cardiac hypertrophy. J Mol Cell Cardiol 49: 334–338. 20433848 10.1016/j.yjmcc.2010.04.008 PMC2885477

[17] Rowe GC, Jiang A, Arany Z (2010) PGC-1 coactivators in cardiac development and disease. Circ Res 107: 825–838. 20884884 10.1161/CIRCRESAHA.110.223818 PMC2955978

[18] Stupp R, Hegi ME, Mason WP, van den Bent MJ, Taphoorn MJ, (2009) Effects of radiotherapy with concomitant and adjuvant temozolomide versus radiotherapy alone on survival in glioblastoma in a randomised phase III study: 5-year analysis of the EORTC-NCIC trial. Lancet Oncol 10: 459–466. 19269895 10.1016/S1470-2045(09)70025-7

[19] Pavlova NN, Thompson CB (2016) The emerging hallmarks of cancer metabolism. Cell Metab 23: 27–47. 26771115 10.1016/j.cmet.2015.12.006 PMC4715268

[20] Yusoff AAM (2015) Role of mitochondrial DNA mutations in brain tumors: A mini-review. J Cancer Res Ther 11: 535–544. 26458578 10.4103/0973-1482.161925

[21] Soon BH, Murad NAA, Then SM, Bakar AA, Fadzil F, (2017) Mitochondrial DNA mutations in grade II and III glioma cell lines are associated with significant mitochondrial dysfunction and higher oxidative stress. Front Physiol 8: 231. 28484394 10.3389/fphys.2017.00231 PMC5399085

[22] Arismendi-Morillo G (2011) Electron microscopy morphology of the mitochondrial network in gliomas and their vascular microenvironment. Biochim Biophys Acta 1807: 602–608. 21692239 10.1016/j.bbabio.2010.11.001

[23] Lamb R, Ozsvari B, Lisanti CL, Tanowitz HB, Howell A, (2015) Antibiotics that target mitochondria effectively eradicate cancer stem cells, across multiple tumor types: Treating cancer like an infectious disease. Oncotarget 6: 4569–4584. 25625193 10.18632/oncotarget.3174 PMC4467100

[24] Wallace DC (2012) Mitochondrial function and cancer. Nat Rev Cancer 12: 685–698. 23001348 10.1038/nrc3365 PMC4371788

[25] William D, Walther M, Schneider B, Linnebacher M, Classen CF (2018) Temozolomide-induced increase of tumorigenicity can be diminished by targeting of mitochondria in in vitro models of patient individual glioblastoma. PLoS One 13: e0191511. 29352318 10.1371/journal.pone.0191511 PMC5774812

[26] Ying M, Sang Y, Li Y, Guerrero-Cazares H, Quinones-Hinojosa A, (2011) Kruppel-like family of transcription factor 9, a differentiation-associated transcription factor, suppresses Notch1 signaling and inhibits glioblastoma-initiating stem cells. Stem Cells 29: 20–31. 21280156 10.1002/stem.561 PMC3516843

[27] Ying M, Tilghman J, Wei Y, Guerrero-Cazares H, Quinones-Hinojosa A, (2014) Kruppel-like factor-9 (KLF9) inhibits glioblastoma stemness through global transcription repression and integrin α6 inhibition. J Biol Chem 289: 32742–32756. 25288800 10.1074/jbc.M114.588988 PMC4239625

[28] Masilamani AP, Ferrarese R, Kling E, Thudi NK, Kim H (2017) KLF6 depletion promotes NF-κB signaling in glioblastoma. Oncogene 36: 3562–3575. 28166199 10.1038/onc.2016.507 PMC5485221

[29] Wan J, Su Y, Song Q, Tung B, Oyinlade O, (2017) Methylated cis-regulatory elements mediate KLF4-dependent gene transactivation and cell migration. Elife 6: e20068. 28553926 10.7554/eLife.20068 PMC5466421

[30] Wang S, Shi X, Wei S, Ma D, Oyinlade O, (2018) Kruppel-like factor 4 (KLF4) induces mitochondrial fusion and increases spare respiratory capacity of human glioblastoma cells. J Biol Chem 293: 6544–6555. 29507094 10.1074/jbc.RA117.001323 PMC5925822

